# Development of an Accelerated Stability Model to Estimate Purple Corn Cob Extract Powder (Moradyn) Shelf-Life

**DOI:** 10.3390/foods10071617

**Published:** 2021-07-13

**Authors:** Lucia Ferron, Chiara Milanese, Raffaella Colombo, Adele Papetti

**Affiliations:** 1 Department of Drug Sciences, University of Pavia, Viale Taramelli 12, 27100 Pavia, Italy; lucia.ferron01@universitadipavia.it (L.F.); raffaella.colombo@unipv.it (R.C.); 2 FlaNat Research Italia Srl, Via Giuseppe di Vittorio 1, 20017 Rho, Milan, Italy; 3 Consorzio interuniversitario per i Sistemi a Grande Interfase & Department of Chemistry, Physical Chemistry Section, University of Pavia, Viale Taramelli, 27100 Pavia, Italy; chiara.milanese@unipv.it

**Keywords:** purple corn cob, anthocyanins, Arabic gum, accelerated stress protocol, forced degradation, moisture-modified Arrhenius equation

## Abstract

Moradyn is an Italian purple corn variety whose cobs represent a rich source of polyphenols. At the industrial level, they are used to produce a dried extract (MCE) by the addition of 20% Arabic gum. In order to evaluate the extract solid-state stability, an innovative accelerated stress protocol was developed following the isoconversion approach. The degradation kinetics of cyanidin-3-*O*-glucoside (C3G), the most suitable marker to monitor the overall MCE degradation status, was monitored under five temperature–humidity (RH) combinations. These data were used to build a mathematical model, able to estimate the C3G stability at 25 °C and 30% RH, whose predictiveness was further assessed by comparing the predicted vs. experimental C3G isoconversion time. Finally, by applying this model, the expiry date of the extract was calculated to be within 26–33 days, confirming that the addition of 20% Arabic gum is insufficient to stabilize MCE and highlighting the need of a new formula in order to prolong MCE shelf-life.

## 1. Introduction

Today people are more aware of the potential health benefits derived from the consumption of nutritionally valuable foods than in previous decades; these are clearly recognized as the primary source of bioactive compounds, claimed not only to have a nutritional function but also to improve health condition by inhibiting typical chronic disease risk factors, such as hyperglycemia, hypertension, obesity, and oxidative stress [1]. Nowadays, a huge number of both epidemiological and in vitro studies support the correlation between the consumption of plant secondary metabolites—such as alkaloids, terpenoids, flavonoids, and phenolic acids—and their bio-protective effects [2]. In particular, flavonoids are known to exert a wide range of different health benefits, including hypoglycemic, hypolipidemic, anti-inflammatory, antimicrobial, and antioxidant effects [3].

In this context, plant-based food supplements (botanicals) represent an interesting approach in the prevention and management of the diseases strictly related to aging and life-style, such as metabolic syndrome [4]. Although researchers and pharmaceutical companies have demonstrated a renewed interest in investigating plants and crops as a source of bioactive phytocomplexes, their application is still limited since the physico-chemical properties and chemical stability of these complex matrices are strongly affected by environmental factors such as pH, temperature, and light [5,6]. Exposure to these factors triggers the degradation pathways of many bioactive metabolites, especially anthocyanins, strongly affecting their storage stability [6].

Over the last five years, interest in the identification of new and effective strategies to prolong the shelf-life of polyphenols and preserve their health value has grown. In particular, research has focused on the use of polysaccharides such as Arabic gum (AG), maltodextrins, or β-cyclodextrin as stabilizing agents. In fact, soluble fibers are able to bind and entrap both hydrophilic and hydrophobic bioactive metabolites (such as lutein, anthocyanins, or piperine) which become less sensitive to environmental factors [7,8,9,10,11,12,13]. Moreover, encapsulation protocols have been designed to optimize stability and bioavailability of molecules present in natural extracts. Maltodextrins and AG are often used as common coating agents, thanks to their high solubility, lack of color and odor, and low cost. AG belongs to a group of water soluble and undigestible polysaccharides which are known to inhibit oxidation reactions and, therefore, are able to protect sensitive compounds when encapsulated [9].

Among flavonoids, anthocyanins have been thoroughly investigated in stability studies because of their extremely low storage stability, especially when affected by high temperatures and pH values higher than 5.0 [5,8]. Different stress testing procedures, based on guidelines reported by the International Council for Harmonisation of Technical Requirements for Pharmaceuticals for Human Use (ICH), the World Health Organization (WHO), and the European Medicines Agency (EMA) [14,15,16], can be applied to verify the stability of a molecule. Stress testing’s main goal is to identify the degradation fate of an active principle ingredient (API), its intrinsic stability, through a validated analytical procedure. Guidance on stability testing of active pharmaceutical ingredients and finished pharmaceutical products, as reported by EMA, states: “The objective of stress testing is to identify primary degradation products and not to completely degrade the API. The conditions studied should cause degradation to occur to a small extent, typically 10–30% loss of API as determined by assay when compared with non-degraded API.” This test should be carried out by submitting a single batch of the API to different temperature (T) and relative humidity (RH) conditions, in order to monitor the effects of these factors on active molecules over time (European Medicines Agency). However, guidelines typically refer to a single bioactive compound, an API, or drugs at the final preparation stage, and not to a natural phytocomplex. Currently, there is a general lack of commitment to pass worldwide effective legislation concerning the evaluation of natural extract quality, chemical stability, and efficacy; however, these features are already mandatory in the production of drugs [11,16,17,18,19]. Compared with well-defined synthetic drugs, plant extract standardization and stability assessment are hard tasks; the high variability of a phytocomplex composition, in addition to all factors influencing the secondary metabolite profile such as the plant’s developmental stage, environmental factors, and post-harvest processing, make standardization feasible, but not easy.

The biological activity of plant-based preparations cannot be attributed to a small number of compounds, but is usually related to the synergistic action of a complex pattern of molecules. Thus, in order to evaluate the quality of the extract, a target group of compounds should be selected and monitored during standardization, as well as during stability studies, following the above-mentioned guidelines [20]. However, the application of these standardized protocols to evaluate a natural extract’s chemical stability is an extremely complex, expensive, and time-consuming procedure.

Recently, our group investigated the chemical composition of an anthocyanin-enriched extract obtained from a new Italian purple corn cob variety, Moradyn (MCE). The phytocomplex of MCE differs in composition from the typical Peruvian purple corn cob in its greater variety of flavonols, such as quercetin, myricetin, isorhamnetin, and kaempferol derivatives, and its lack of malonylated anthocyanins, which are typically detected in purple corn varieties. MCE demonstrated good hypoglycemic and antiglycative activities in several in vitro experiments, attributed to the synergistic action of all polyphenols present in the extract [21]. Therefore, MCE could be a good candidate for a healthy formulation, but only after stability studies.

Therefore, the aim of this work was to assess MCE storage stability, when AG is used as a carrier agent, by applying an innovative statistical approach. In the current study, stress testing was carried out using a new solid-state stability model based on the accelerated stability assessment program (ASAP) which allowed for evaluation of the effect of temperature and humidity on MCE and prediction of its storage stability [22,23].

## 2. Materials and Methods

### 2.1. Chemicals

Ethanol, magnesium chloride, sodium chloride, HPLC-grade formic acid, and acetonitrile were purchased from Carlo Erba (Milan, Italy). Water was obtained from a Millipore Direct-QTM system (Merck-Millipore, Milan, Italy). Arabic gum was purchased from Merck Life Science (Milan, Italy). Kuromanin chloride (cyanidin-3-*O*-glucoside) was purchased from Extrasynthese (Genay, Rhone, France).

### 2.2. Moradyn Corn Cob Extract (MCE) Preparation

Chopped Moradyn corn cobs (Community Plant Variety Office Registration-Examination Ref. 4067062) were provided by FlaNat Research Italia S.r.l. (Milan, Italy), and extracted following the procedure described in [21]. MCE dry matter was then suspended in AG aqueous solution (4:1, *w*/*w*), and dried using a vacuum drying oven at 40 °C for 48 h.

### 2.3. Stress Conditions and Accelerated Stability Model Validation

Stress tests were performed by submitting MCE-AG dry matter to five different storage conditions: 25 °C at 75% RH, 45 °C at 30 and 75% RH, and 70 °C at 30 and 75% RH. Twenty milligrams of dried MCE-AG were weighed in a 15 mL open glass vial, inserted into a sealed vessel containing saturated salt solutions, in order to maintain a controlled relative humidity value in the chamber, and then stored in a controlled temperature oven. Saturated magnesium chloride and sodium chloride solutions were used to maintain the environment at 30% RH and 75% RH, respectively [24].

In order to validate the mathematical model, three MCE-AG samples from three different batches were prepared following the above-mentioned protocol. Three different samples obtained from each batch were submitted to stress testing at 45 °C-30% RH, 45 °C-75% RH, and 70 °C-75% RH, and their degradation was assessed by monitoring cyanidin-3-*O*-glucoside (C3G) concentration by HPLC at three different times (4, 5, and 7 days for 45 °C-30% RH; 4 h, 1 day, and 4 days for 45 °C-75% RH; 2 h, 5 h, and 1 day for 70 °C-75% RH). All chromatograms were recorded both at 520 nm and 370 nm in order to monitor the changes occurring in anthocyanin and flavonoid concentrations.

### 2.4. Chemical Characterization by RP-HPLC-WVD

An Agilent Technologies 1260 Infinity high-performance liquid chromatography system (Santa Clara, CA, USA), which included a quaternary gradient pump, an autosampler, a degasser, and a variable wavelength detector (VWD), was used. The chromatographic separation was performed using a Gemini C18 analytical column (150 × 2.0 mm i.d., 5 μm, Phenomenex, Torrance, CA, USA) thermostatted at 25.0 ± 0.5 °C, operating at a constant flow rate of 0.3 mL/min, injection volume 20 μL. The mobile phase consisted of 0.1% formic acid aqueous solution (solvent A) and acetonitrile acidified with 0.01% formic acid (solvent B); the gradient elution was: 0–3 min, 2–15% B; 3–45 min, 15–25% B; 45–48 min, 25–35% B; 48–58 min, 2% B, followed by a column reconditioning step of 10 min. Chromatograms were recorded at 370 and 520 nm. The HPLC-VWD system was controlled by Agilent OpenLab CDS ChemStation software (Windows 10, Agilent Technologies, Santa Clara, CA, USA).

#### RP-HPLC-WVD Method Validation

The validation tests were carried out using the external standard method, following ICH guidelines on bioanalytical method validation [25]. C3G was used as standard and chromatograms were registered at 520 nm. In order to verify a putative matrix effect, C3G was dissolved both in MCE and in acidulate aqueous-acetonitrile solution at 100 µg/mL. Both solutions were used to construct two different five-point calibration curves in the 5–20 µg/mL concentration range; each point was analyzed in triplicate and R^2^ values were compared.

Specificity was assessed by overlapping chromatograms obtained for C3G and blank solution (mobile phase) and no peak was detected in the latter; selectivity was verified by comparing the retention time of standard reference C3G in sample solvent to the retention time of C3G in MCE, recorded at 520 nm.

As regards linearity, three calibration curves (in three non-consecutive days) in the range 5–20 µg/mL were constructed for C3G by plotting the integrated peak area (y) at five different concentration levels vs. the theoretical concentration.

Limit of detection (LOD) and limit of quantification (LOQ) were determined by the signal-to-noise ratio (S/N) and defined as the concentration levels at S/N of about 3 and 10, respectively. The precision of the method was evaluated by intra- and inter-day estimation, repeating the analysis of the highest tested concentration (20 µg/mL) nine times within a single day (intra-day) and, in triplicate, three different concentrations (5, 10, and 20 µg/mL) for three consecutive days (inter-day). Finally, the precision was measured by computing the relative standard deviation (RSD%) on replicate analyses, while accuracy was assessed by recovery studies of C3G at three different concentration levels (5, 10, and 20 µg/mL), performed in triplicate.

### 2.5. Solid-State Characterization

Differential scanning calorimetry (DSC) analyses were performed by heating the samples (about 5 mg) from −80 °C to 300 °C at 5 °C/min under N_2_ atmosphere in open Al crucibles in a Q2000 instrument interfaced with a TA 5000 data station (TA Instruments, USA) and subsequently cooling them back to room temperature.

Fourier-transform infrared (FT-IR) spectra were acquired using a Nicolet FT-IR iS10 Spectrometer (Nicolet, Madison, WI, USA) equipped with an attenuated total reflectance (ATR) sampling accessory (Smart iTR with diamond plate) by co-adding 32 scans in the 4000 cm^−1^–650 cm^−1^ range with resolution set at 4 cm^−1^.

### 2.6. Statistical Analysis

Matlab statistical software version R2019B was used to build the solid-state stability model, based on moisture-corrected Arrhenius equation, and to perform ANOVA tests.

## 3. Results and Discussion

MCE is a rich source of polyphenols and its anthocyanin fraction represents 28% (*w*/*w*) of its total phenolic content [21]. Anthocyanins are well known for their health benefits; however, their use is still limited by their sensitivity to pH, light, oxygen, and heat, which lead to weak storage stability [26]. In order to increase anthocyanins’ storage stability, different formulations developed by coupling these compounds with different wall materials, such as maltodextrins [27] or Arabic gum, are present in the literature. In particular, Arabic gum could be a suitable anthocyanin stabilizer when drying techniques are used to obtain the final ingredient [28]. Therefore, in this study, MCE was formulated with 20% AG (*w*/*w*) (MCE-AG), which is a concentration generally used at the industrial manufacturing level to produce a dried extract.

### 3.1. HPLC Validation

The RP-HPLC method was validated according to ICH guidelines on bioanalytical method validation [25]. Specificity, selectivity, linearity, limit of detection (LOD), limit of quantification (LOQ), precision (intra- and inter-day), and accuracy were evaluated.

Preliminary experiments demonstrated that no matrix effect was registered, thus all the analyses were carried out using C3G dissolved in 0.1% formic acid aqueous solution—acetonitrile acidified with 0.01% formic acid, 80:20 (*v*/*v*).

Method specificity was assessed by overlapping chromatograms obtained for C3G at the concentration of 20 µg/mL and blank solution (mobile phase) and no peak was detected in the latter, while selectivity was verified by comparing the retention time of the standard with the retention time of C3G detected in MCE, recorded at 520 nm. Over three different days, a calibration curve (5–20 µg/mL) was freshly prepared, analyzed, and linear regression analysis was performed using the least-squares method. The resulting coefficient of correlation mean value of these three curves was higher than 0.998, indicating a good linearity.

The method was shown to be accurate, with values ranging from 99.648 to 101.604%, and precise, since all standard deviation values were lower than 0.3%. Finally, the sensitivity was assessed by calculating LOD and LOQ values, which were 0.925 µg/mL and 3.082 µg/mL, respectively.

Therefore, overall, the data demonstrated that this analytical method is suitable to quantify C3G in MCE.

### 3.2. Preliminary Physico-Chemical Characterization of the Raw Materials and Their Mixtures

Preliminary thermal analysis (DSC) and IR measurements were performed on MCE, C3G, AG, and their mixture to evaluate if 20% AG (*w*/*w*) could modify MCE and C3G when exposed to high temperatures.

**Figure 1:**
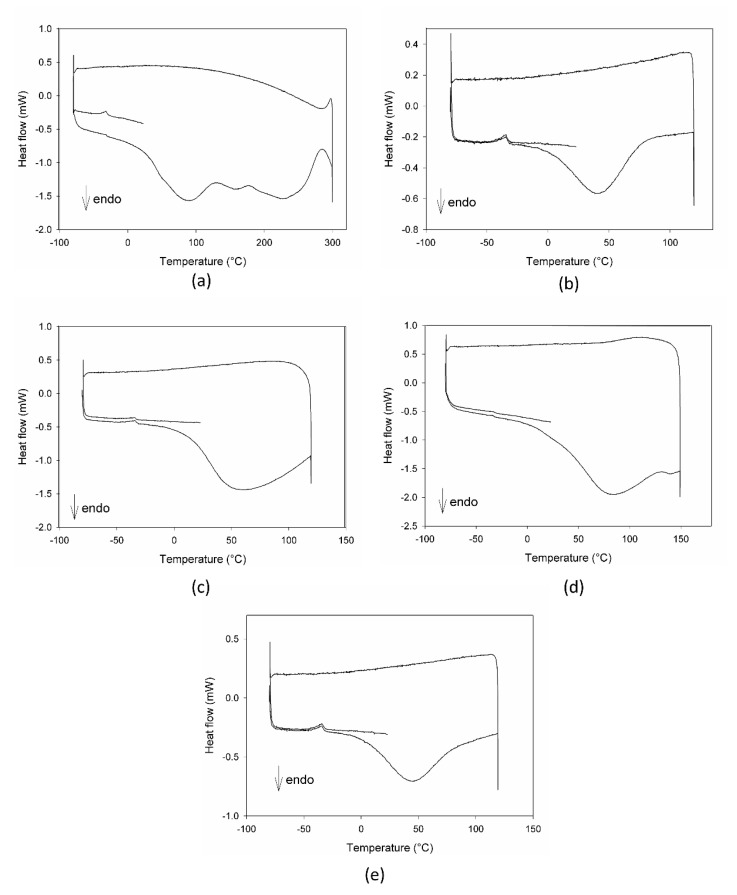
DSC profiles of Moradyn corn cob extract-MCE (**a**), cyanidi-3-*O*-glucoside-C3G (**b**), Arabic gum-AG (**c**), mixture MCE-20% AG, *w*/*w* (**d**), and mixture C3G-20% AG, *w*/*w* (**e**).

**Figure 2:**
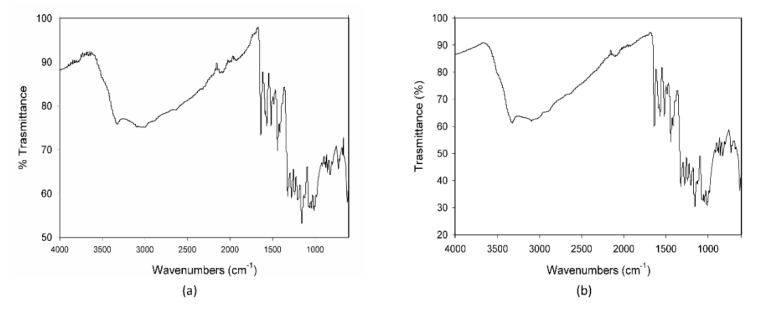
IR spectra of pure cyanidin-3-O-glucoside-C3G (**a**) and of the mixture C3G-20% AG, *w*/*w* (**b**).

The calorimetric profiles acquired for the different substances are reported in Figure 1. In the range 80–300 °C, MCE presented three large endothermic peaks (Figure 1a), centered, respectively, at 90 °C, 160 °C, and 225 °C, due to the decomposition of some of its different constituents, as proven by the fact that the cooling curve showed no peaks. The first peak appeared as the superimposition of two processes with different kinetics, the first one, faster, starting at around 0 °C and the second one, slower, starting at about 50 °C. C3G (Figure 1b) showed only one sharper endothermic peak, starting at about 0 °C and centered at 40 °C, under which the substance decomposed. This peak resembled the features of the first signal in MCE, indicating that C3G could be the first component decomposing and hence the most instable of the MCE constituents. For this reason, C3G was chosen as a marker of the stability of the MCE mixtures, and its decomposition temperatures as the best temperature range to study the behavior of the mixtures under thermal stress. The Arabic gum (Figure 1c) showed a very large and asymmetric signal starting at −35 °C, centered at 60 °C and not ended by 110 °C, due to irreversible processes happening in the matrix. The calorimetric curves of the mixtures MCE–20% AG (*w*/*w*) (Figure 1d) and C3G-20% AG (*w*/*w*) (Figure 1e) were superimposable in shape and characteristic temperatures, up to 150 °C, to those of the main components (Figure 1a,b, respectively), with no effect and by-side reactions attributable to the gum presence in the examined temperature range. To further demonstrate the compatibility between C3G and Arabic gum, IR spectra of the pure component and of the 20% mixture were acquired. As evident in Figure 2a,b, the two graphs were superimposable and all the bands and peaks resulted attributable to the C3G functional groups.

### 3.3. Stability Assessment Model

MCE stability was estimated by conducting a new isoconversion approach based on the accelerated stability assessment program [22,23], usually applied to drugs and never, to the best of the authors’ knowledge, to a natural product consisting of many compounds.

Bioactive molecules’ degradation behavior, when submitted to high temperature and humidity levels, is typically described by a moisture-corrected Arrhenius equation [23],

lnK = lnA + [Ea/(R × T)] + [(B × %RH)](1)

where K is the degradation rate (degradation %/day), A is the collision frequency factor, T is temperature (K), R is the gas constant, RH is relative humidity, and Ea and B are the activation energy and humidity sensitivity constant related to the studied compounds, respectively.

Temperature and relative humidity are the main drivers of instability, however K is also related to time, as the registered reaction rates typically decrease with the extent of the conversion of reacting compounds. This is due to the fact that all of the compounds present in a mixture, at the solid state, coexist in multiple states, each characterized by a different sensitivity to environmental conditions. When complex mixtures are exposed to stressful conditions, each state reacts with its own kinetics, based on T or RH levels. During the degradation process, quantities and ratios between these different states are continuously changing, influencing the overall kinetics and degradation rate constant. Therefore, if the degradation is monitored at fixed time points independently from T and RH values, the degradation rates extrapolated from the so obtained dataset cannot be predictive of degradation rates occurring under different environmental conditions. In fact, the overall composition of each collected sample at a fixed time point is characterized by a unique pattern of states, based on T or RH, which cannot be compared with the one obtained at the same time point but under different conditions.

Following the isoconversion approach, degradation state is no longer monitored at multiple, fixed times, but is instead determined considering only the isoconversion time, i.e., the period required by the selected marker compound or API to reach the specification limit of degradation when exposed to a specific storage condition, which varies based on temperature and humidity values.

During stress testing, the selected specification limit should be within the range of 5–20% degradation of the selected markers [14,29], as it would lie in the first degradation stage, which generally follows a linear behavior [30]. Moreover, this method allowed for the mathematical prediction of the isoconversion time at any T and RH combination from Equation (1), knowing the three terms, A, Ea, and B, related to the marker compound. In fact, when the sample reached the specification limit of degradation, the K constant received the same contribution from each state, independently of T and RH levels [22].

Therefore, in order to extrapolate A, Ea, and B terms related to MCE, this extract was submitted to five different combinations of T and % RH (Table 1). Its degradation was monitored by assessing the gradual decrease of C3G, the marker selected as it is the most abundant anthocyanin in MCE [21], at four different time points, as reported in Table 1, and the specification limit was selected considering the highest degradation value lies on the first linear stage of the reaction.

Then, the experimental degradation rate for each T and RH combination, expressed as lnK value, was extrapolated from the curve obtained by plotting % C3G reduction vs. time, and it corresponded to the slope of the straight line passing from the origin and the point related to the chosen specification limit [29]. By using these experimental lnK values, a multiple regression model was built and the three terms of the Arrhenius equation were extrapolated. These extrapolated terms were used to realize the construction of a predictive mathematical model, based on the Arrhenius equation [22], which was applied to calculate MCE isoconversion time at 25 °C and 30% RH, aiming to approximate its expiry date.

#### 3.3.1. Degradation Kinetics

**Figure 3:**
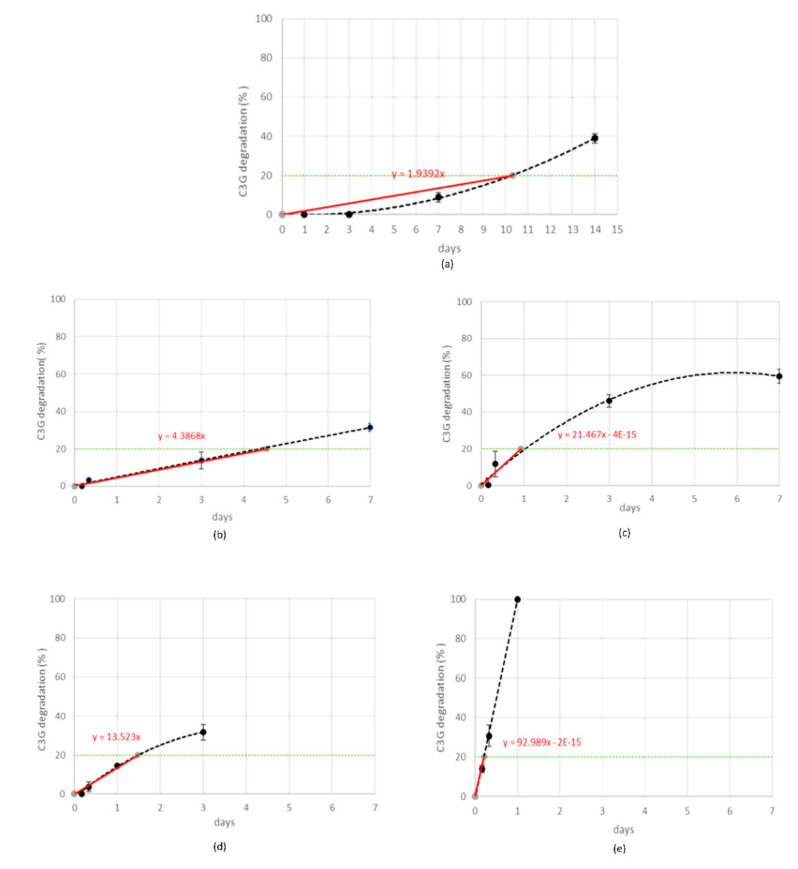
Cyanidin-3-*O*-glucoside (C3G) in MCE degradation kinetics (black line) when exposed to 25 °C-75% RH (**a**), 45 °C-30% RH (**b**), 45 °C-75% RH (**c**), 70 °C-30% RH (**d**), or 70 °C-75% RH (**e**). Experimental rate constants (ExpK) for each treatment were determined from the slope of the straight line (red line) passing from the origin and the specification limit (green line).

Stress tests were performed by submitting MCE to five different storage conditions and monitoring the C3G reduction percentage (quantified by RP-HPLC-WV) at fixed time points, as reported in Table 1. The gradual C3G reduction was followed for each stress condition and the order of reaction kinetics was graphically determined by choosing the fitting which gave the best correlation coefficient (Figure 3) [31].

**Figure 4:**
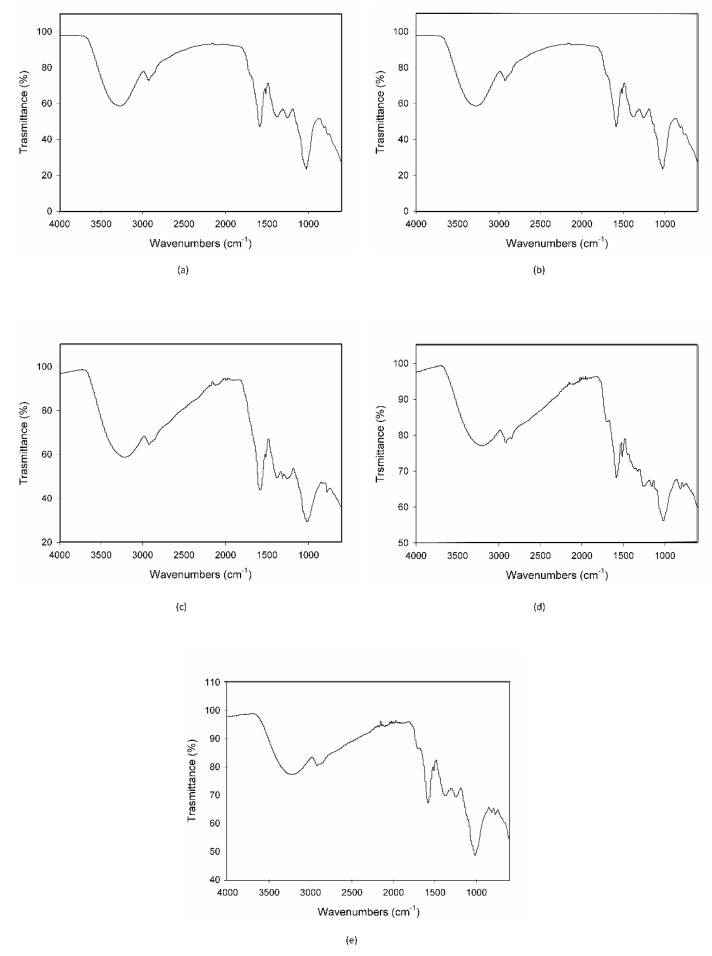
IR spectra of the MCE-20% AG (*w*/*w*) after 24 h of degradation at 70 °C-75% RH (**a**), after 4 h (**b**) and 3 days of degradation at 70 °C-30% RH (**c**), and after 3 (**d**) and 7 days of degradation at 25 °C-75% RH (**e**).

When MCE was exposed to the strongest condition, (70 °C-75% RH), C3G totally degraded during the first 24 h, following zero-order kinetics, and therefore its degradation was not dependent on its initial concentration (Figure 3e). This is demonstrated also by the IR spectroscopy; the acquired spectrum for this sample was totally different from those before treatment (Figure 2b and Figure 4a). On the other hand, when samples were submitted to mild conditions (45 °C-75% RH and 70 °C-30% RH), two different steps were identified: in the first part of the reaction the degradation rate was faster, while it decreased step by step in the last phase (Figure 3c,d), following the classical degradation kinetic already stated by Waterman [22]. This behavior could be due to an initial faster degradation of free C3G which was highly exposed to oxygen action, followed by a slower degradation of bounded anthocyanin fraction, in accordance with the results reported by Tonon for açai juice [13]. The IR spectra for the samples treated for 4 h and 3 d at 70 °C-30% RH are reported in Figure 4b,c; after only 4 h of treatment the characteristic features of the functional groups of C3G were almost totally lost and after 3 days the spectrum was almost superimposable to the spectrum of a totally degraded sample (Figure 4a). This trend was not observed at 45 °C-30% RH (Figure 3b), but a longer monitoring period is likely required and more time points should be evaluated to better investigate degradation kinetics under this condition. When the degradation rate was monitored in the presence of high humidity at room temperature (25 °C and 75% RH), the reaction had an opposite trend (Figure 3a). In fact, anthocyanins are known to be extremely sensitive to high temperatures, but high humidity levels could also contribute to increases in their instability by accelerating chemical and physical degradations [9,13]. Thus, at room temperature and high humidity values, C3G degradation is only delayed and gets faster after three days of exposure to such conditions (see IR spectra in Figure 4b,e for 3 days and 7 days aging).

**Figure 5:**
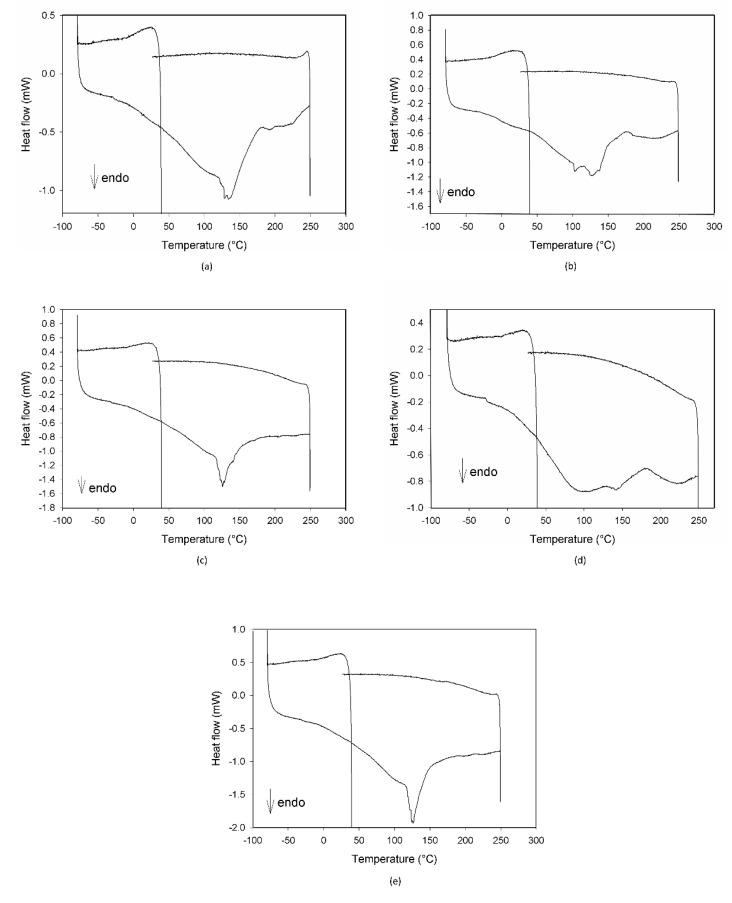
DSC profiles of the mixture MCE-20% AG (*w*/*w*) after 24 h of degradation at 70 °C-75% RH (**a**), after 4 h (**b**) and 3 days of degradation at 70 °C-30% RH (**c**), and after 3 (**d**) and 7 days of degradation at 25 °C-75% RH (**e**).

In Figure 5, the DSC profiles of the same samples discussed for the IR results are reported. As evident in all of the plots, no signals attributable to C3G are present, with the exception of a small shoulder in Figure 5b, confirming its degradation due to ageing.

Both the DSC and IR plots also demonstrated the gradual degradation of the different components of the mixtures, with a different evolution depending on the ageing conditions.

#### 3.3.2. Determination of Ea, lnA, and B Arrhenius Equation Terms and Development of a Mathematical Model

As mentioned above, during stress testing it is recommended to select the specification limit of degradation within the range of 5–20% degradation of the monitored marker [14,29], as it would lie in the first degradation stage which typically follows a linear behavior [30].

**Figure 6:**
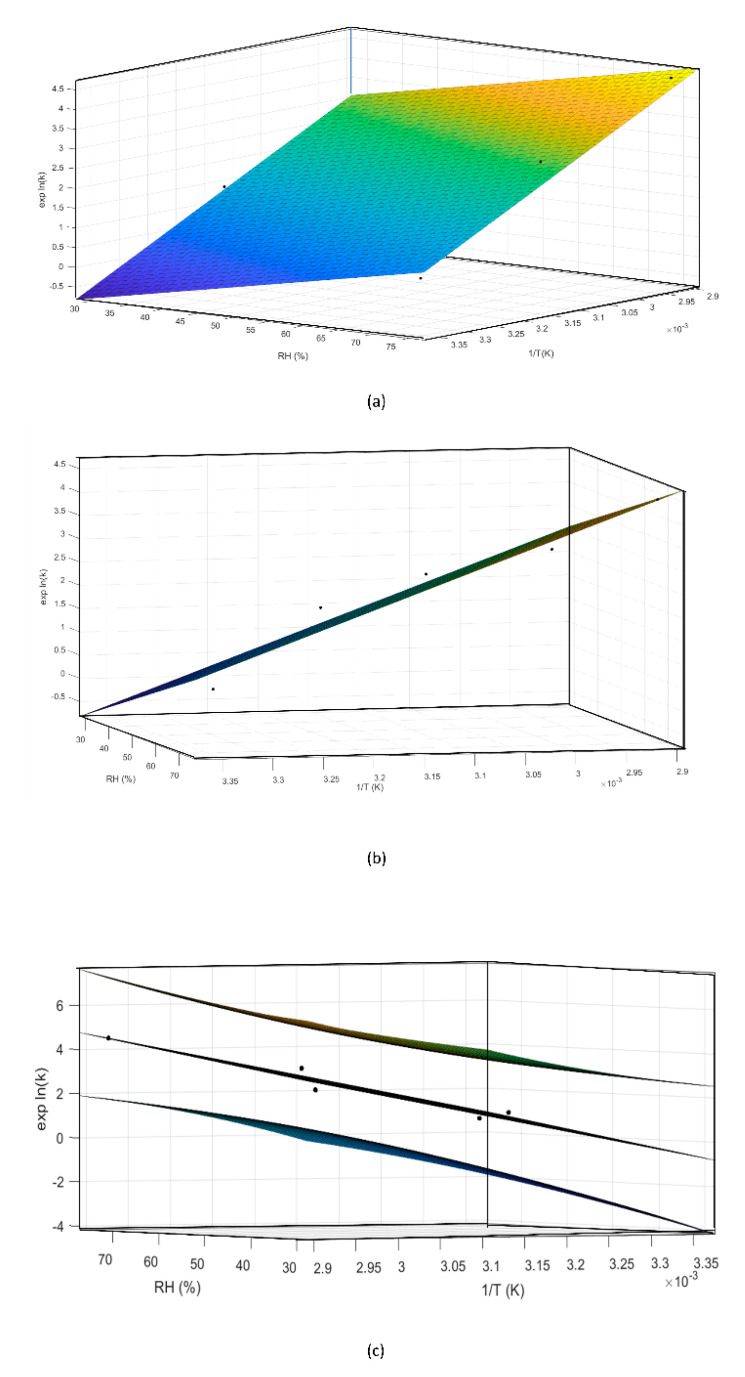
Different perspectives of a three-dimensional surface plot of the model generated by the Matlab curve-fitting tool (**a**,**b**). Using the same tool the confidence bounds for this fitting were calculated with a level of certainty of 95%, represented by the upper and lower planes (**c**).

During preliminary stress testing, MCE was exposed to high temperature values (both in the presence and absence of high humidity values), and C3G degradation kinetics between 0% and 20% could always be simplified and approximated as zero order (Figure 3), therefore 20% was selected as the specification limit to be used during the ASAP protocol. Thus, based on the isoconversion concept, the experimental rate constant (exp K), expressed as degradation % per day, was derived for each tested condition from the slope of straight lines passing from the origin of graphs and the point corresponding to 20% degradation, calculated by interpolation of each curve (Figure 3a–e, red lines). The natural logarithms of all five exp K (exp lnK) were fit with the reciprocal of temperature (1/T (K)) and relative humidity (RH (%)), using the Matlab multiple regression function, to extrapolate Ea, lnA, and B terms related to C3G. Then, the Matlab curve-fitting tool was applied to graphically represent this model (Figure 6a,b). Following ASAP assumption, the surface slope represented lnA, while angles determined by the surface and 1/T and RH % axes corresponded to Ea and B, respectively.

Finally, a mathematical model was constructed by fitting Equation (1) to the experimental data:

lnK = 24.209 − [(7626.6 × 1/T) + (0.033 × RH%)](2)

**Figure 7:**
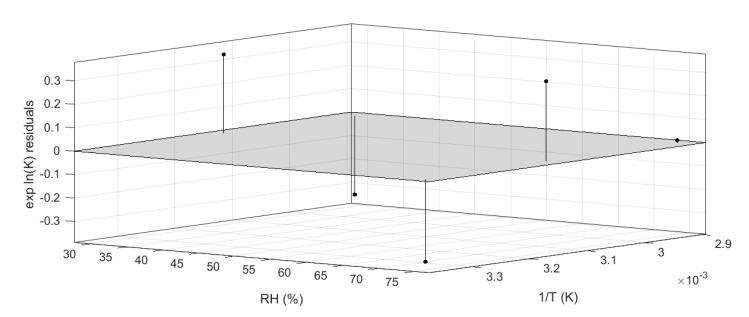
Residual plot generated by Matlab curve-fitting tool.

This model was described by an adjusted-R squared of 0.94, and all residuals were properly distributed within the 95% confidence interval, as shown in Figure 7. The accuracy of the regression was estimated by calculating the coefficient of variation of the root mean squared error (which is a not-scaled dependent factor) and it was found to be 3.1% [32,33].

The quality of this model was further evaluated by the application of ANOVA analysis (α = 0.05), which highlighted the significance of the regression with a *p*-value of 0.0063.

**Figure 8:**
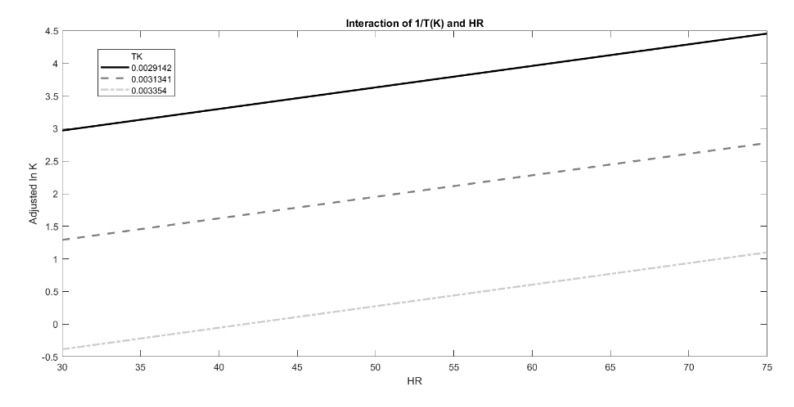
Interaction plot of the effect exerted by growing RH % on lnK at fixed T values (1/T (K)), represented by the different lines.

However, the effect of T and RH could be modeled on lnK following different approaches in order to find the best fit with experimental data (e.g., adopting other degradation shape parameters, or introducing new interaction terms to Equation (1), especially when no linear relation between the observed degradation rate and time is present.) Thus, in order to investigate the possibility of further improvement of the fit, the effects of the two predictors, 1/T and RH%, on lnK values and their interactions were also graphically estimated using the interaction plot analysis (Figure 8). Temperature had a positive effect on lnK values, which increased with lower 1/T values, as shown in Figure 8. Moreover, humidity exerted an additive effect on temperature, as evident from a positive slope of the three curves. However, the interactions between these two predictors were not meaningful, confirming the proper use of the original moisture-corrected Arrhenius equation.

The so-constructed mathematical model described C3G behavior when exposed to stressful conditions.

Moreover, this model can be further used to extrapolate C3G lnK values and predict the isoconversion time for each temperature–humidity combination. Hence, applying this model, it is possible to calculate the C3G isoconversion time at 25 °C-30% RH, the condition used to mimic typical storage conditions [1]). By applying the model (Equation (2)), it was predicted that C3G could decrease to its specification limit after a period of between 26 and 33 days of storage.

### 3.4. Mathematical Model Validation

Further experiments were performed in order to assess the predictiveness of this mathematical model and thus validate it. The model (Equation (2)) was applied to extrapolate the C3G isoconversion times (using 20% degradation as a specification limit) for an intermediate temperature with high or low humidity values (45 °C-75% RH, 45 °C-30% RH, respectively) or the strongest condition tested (70 °C-75% RH).

Then, three different samples obtained from different independent batches of MCE were submitted to these selected stress conditions. Furthermore, degradation state was predicted at two additional time points for each tested condition, respectively before and after the isoconversion time, to better investigate the predictive potential of this model. At the end of each treatment, the degradation rate was assessed and the deviation of the predicted degradation from experimental values were calculated for all time points and conditions tested (Table 2). The main disagreement deviance (24%) was observed at 45 °C-75% RH after 1 day of storage, when the estimated degradation was 20%, but the experimental degradation was 28.44% ± 0.25. This difference was probably due to the fact that, at mild temperatures, degradation is strongly driven by humidity, whose effect cannot be fully described by a linear fit, as previously mentioned.

However, considering the overall result, the model demonstrated a satisfactory predictive capability with an average fitting deviation of 11.87% (calculated as the mean of the deviations observed at each tested condition).

### 3.5. MCE Quality Assessment

Considering that MCE is a phytocomplex characterized by the presence of a great variety of flavonols such as quercetin, myricetin, isorhamnetin, and kaempferol derivatives, and that its healthy properties are correlated to the whole phytocomplex [21], any stability assessment study of MCE has to also include flavonols. This became mandatory in order to perform a comprehensive quality control study. However, application of the ASAP approach to each polyphenol present in MCE would require too long a time and was thus unsuitable for industrial scale-up. Therefore, a smart approach could be to monitor the flavonols’ degradation states at C3G degradation isoconversion time when exposed to different T and RH% conditions. The reduction percentage of the five most abundant flavonols (myricetin-3,7-di-*O*-hexoside, quercetin-7-*O*-glucoside, kaempferol-7-*O*-(6”-*O*-malonyl)-hexoside, isorhamnetin-7-O-rutinoside, and isorhamnetin-3-*O*-hexoside) and the other two anthocyanins (perlagonidin-3-*O*-glucoside and peonidin-3-*O*-glucoside) present in MCE was monitored at the predicted C3G isoconversion times (120 h, 24 h, 5 h) under the three selected T and RH% conditions (45 °C-30% RH, 45 °C-75% RH, and 70 °C-75% RH) for evaluation of the stability quality of MCE.

The results obtained by monitoring the reduction percentage of the eight markers from three different MCE batches at 520 and 370 nm are reported in Table 3. Perlagonidin-3-*O*-glucoside followed a degradation trend similar to that of C3G, while peonidin-3-*O*-glucoside reached a higher degradation level at all monitoring conditions. On the other hand, the degradation of each flavonol never reached 20%, with the exception of those stored at the most extreme conditions applied (70 °C–75% RH). However, under these conditions the overall degradation percentage was very similar to that reached by C3G.

## 4. Conclusions

Before now, to our knowledge, stability studies of natural extracts have never been conducted using the isoconversion approach, even though this methodology allows for estimation of the degradation entity in a shorter time, and with better accuracy, than the classical protocol.

Our approach allowed us to estimate the storage stability of a natural extract, consisting of a large number of compounds, without generating an enormous dataset. In fact, by selecting the marker compound which was the most sensitive to degradation parameters and extrapolating its isoconversion time, it was possible to predict the overall phytocomplex shelf-life. In the current study, C3G was shown to be one of the most sensitive compounds in MCE as it reached the specification limit (20%) faster than the other compounds. For this reason, it was selected as a marker in order to monitor MCE degradation. Therefore, considering the isoconversion time of C3G at 25 °C-30% RH (which mimics ambient storage conditions) as a reference for the prediction of the storage stability of the entire phytocomplex, the shelf-life range of MCE-20% Arabic gum (*w*/*w*) formulation was estimated to be between 26 and 33 days.

In conclusion, the application of this new approach to estimate the stability of anthocyanin enriched-extracts could represent an interesting strategy and may be particularly useful for rapidly screening different formulations with the aim of optimizing the solid-state stability of such products. As a result of this work, the MCE-20% Arabic gum (*w*/*w*) formulation’s storage stability was found to be inappropriate for market purposes, and thus a future perspective will include further stress tests performed using different Arabic gum concentrations and considering the effect of oxygen and light in order to prolong MCE shelf-life.

## Figures and Tables

**Table 1:** Conditions used for forced degradation tests and experimental scheme applied.

Storage Condition	Monitoring Times
25 °C-75% RH	0 d, 1 d, 3 d, 7 d, 14 d
45 °C-30% RH	0 h, 4 h, 8 h, 3 d, 7 d
45 °C-75% RH	0 h, 4 h, 8 h, 3 d, 7 d
70 °C-30% RH	0 h, 4 h, 8 h, 1 d, 3 d
70 °C-75% RH	0 h, 4 h, 8 h, 1 d, 3 d

**Table 2:** Cyanidin-3-O-glucoside (C3G) relative degradations observed after each treatment at the monitored times and their deviation percentage (Dev %) from the predicted values. Average deviations (Ave dev) were calculated for each condition. Exp Degradation was calculated as the mean of all degradation values registered for the three tested batches.

Condition	Time (h)	Exp Degradation (%) ± DS	Predicted Degradation (%)	Dev (%)	Ave Dev (%)
70 °C-75% RH	2	5.311 ± 1.67	7.10	11.51	6.27
	5	18.953 ± 7.38	20.46	5.41	
	24	87.553 ± 0.54	85.25	1.89	
45 °C-75% RH	4	4.194 ± 0.825	3.35	15.86	13.72
	24	28.444 ± 0.255	20.09	24.34	
	96	79.292 ± 3.35	80.36	0.95	
45 °C-30% RH	96	15.841 ± 5.67	15.52	1.45	3.2
	120	18.237 ± 6,66	19.40	4.37	
	168	28.651 ± 0.896	27.16	3.78	

**Table 3:** Peak area relative reduction (%) of selected marker compounds present in MCE, monitored at the estimated isoconversion times (120 h, 24 h, and 5 h) for each treatment (45 °C-30% RH, 45 °C-75% RH, and 70 °C-75% RH). All values were calculated as the mean of the relative reductions observed for the three tested batches.

Compound	Reduction (%)		
	45 °C-30% RH (120 h)	45 °C-75% RH (24 h)	70 °C-75% RH (5 h)
cyanidin-3-*O*-glucoside	21.70 ± 1.66	28.72 ± 0.16	19.13 ± 6.08
perlagonidin-3-*O*-glucoside	20.78 ± 2.77	25.59 ± 6.99	14.57 ± 5.7
peonidin-3-*O*-glucoside	30.70 ± 3.9	30 ± 1.07	34.45 ± 4.49
myricetin-3,7-di-O-hexoside	0	8.44 ± 1.89	3.02 ± 2,66
quercetin-7-*O*-glucoside	0	0	0
kaempferol-7-*O*-(6″-*O*-malonyl)-hexoside	0	1.59 ± 0.99	27.15 ± 4.71
isorhamnetin-7-*O*-rutinoside	0	0	12.96 ± 1.14
isorhamnetin-3-*O*-hexoside	7.41 ± 3.83	0	29.18 ± 10.63

## Data Availability

All data used in the study are available in the present article.
